# Assessment of COVID-19–Related Immigration Concerns Among Latinx Immigrants in the US

**DOI:** 10.1001/jamanetworkopen.2021.17049

**Published:** 2021-07-19

**Authors:** Carol L. Galletly, Julia Lechuga, Julia B. Dickson-Gomez, Laura R. Glasman, Timothy L. McAuliffe, Iván Espinoza-Madrigal

**Affiliations:** 1 Center for AIDS Intervention Research, Department of Psychiatry and Behavioral Medicine, Medical College of Wisconsin, Milwaukee; 2 Department of Public Health Sciences, College of Health Sciences, University of Texas–El Paso, El Paso; 3 Institute for Health Equity, Medical College of Wisconsin, Milwaukee; 4 Lawyers for Civil Rights, Boston, Massachusetts

## Abstract

**Importance:**

Despite the contentious immigration environment and disproportionate rates of COVID-19 infection among Latinx individuals in the US, immigrants’ concerns about engaging in COVID-19–related testing, treatment, and contact tracing have been largely unexplored.

**Objective:**

To examine the proportions of Latinx immigrants who endorse statements about the potential negative immigration ramifications of seeking and using COVID-19–related testing and treatment services and engaging in contact tracing.

**Design, Setting, and Participants:**

In this cross-sectional survey study, 25 COVID-19–related items were incorporated into the online Spanish-language survey of an ongoing study. Data were collected between July 15 and October 9, 2020, in Chicago, Illinois; Los Angeles, California; and Phoenix, Arizona. A nonrandom sample of 379 adult, Spanish-speaking, noncitizen Latinx immigrants (with either documented or undocumented immigration status) were sent surveys. Of those, 336 individuals (88.7% participation rate) returned surveys, and 43 individuals did not. An additional 213 individuals were screened but ineligible. Descriptive statistics were computed, and mean comparisons and bivariate correlations between sociodemographic variables, indices of immigration risk, and COVID-19–related survey items were conducted.

**Main Outcomes and Measures:**

Items elicited agreement or disagreement with statements about immigrants’ access to COVID-19–related testing and treatment services and the potential immigration ramifications of using these services. Willingness to identify an undocumented person during contact tracing was also assessed.

**Results:**

A total of 336 Latinx immigrants completed surveys. The mean (SD) age of participants was 39.7 (8.9) years; 210 participants (62.5%) identified as female, and 216 participants (64.3%) had undocumented immigration status. In total, 89 participants (26.5%) agreed that hospital emergency departments were the only source of COVID-19 testing or treatment for uninsured immigrants, and 106 participants (31.6%) agreed that using public testing and health care services for COVID-19 could jeopardize one’s immigration prospects. A total of 96 participants (28.6%) and 114 participants (33.9%), respectively, would not identify an undocumented household member or coworker during contact tracing. Reluctance to identify an undocumented household member or coworker was associated with having had deportation experiences (*r* = −0.17; 95% CI, −0.06 to 0.27; *P* = .003) but not with the number of years lived in the US (*r* = 0.07; 95% CI, −0.16 to 0.17; *P* = .15) or immigration status (*r* = 0.03; 95% CI, −0.07 to 0.13; *P* = .56).

**Conclusions and Relevance:**

In this cross-sectional survey study, a substantial number of immigrants endorsed statements about immigrants’ restricted access to COVID-19–related testing and treatment services and the potential negative immigration ramifications of using these services. These results suggest that programs for COVID-19–related testing, contact tracing, and vaccine administration that are designed to allay immigration concerns are needed.

## Key Points

**Question:**

What proportions of Latinx immigrants endorse statements about the immigration ramifications of engaging in COVID-19–related testing, treatment, and contact tracing?

**Findings:**

In this survey study of 336 adult Latinx immigrants in the US, 89 participants (27%) believed that hospital emergency departments provided the only source for COVID-19–related testing or treatment for uninsured immigrants. A total of 106 participants (32%) agreed that using public COVID-19–related testing and treatment services could jeopardize an individual’s immigration prospects; 96 participants (29%) and 114 participants (34%), respectively, would not identify an undocumented household member or coworker during contact tracing.

**Meaning:**

These results suggest that substantial proportions of Latinx immigrants have immigration concerns about engaging in COVID-19–related testing, treatment, and contact tracing.

## Teaser

This cross-sectional survey study examines the proportion of adult Latinx immigrants in the US who endorse statements about the immigration ramifications of engaging in COVID-19–related testing, treatment, and contact tracing.

## Introduction

Studies suggest that immigration concerns deter Latinx immigrants from seeking and using medical diagnostic and treatment services.^1,2,3,4^ Despite disproportionate rates of COVID-19 morbidity and mortality among Latinx individuals relative to other population subgroups^5,6^ and the contentious immigration environment in the US, Latinx immigrants’ concerns about seeking and using COVID-19–related testing and treatment services and their willingness to engage in contact tracing have been largely unexamined. We conducted a cross-sectional survey study to begin to explore these knowledge gaps.

## Methods

A total of 25 COVID-19–related items were incorporated into the cross-sectional online survey of an ongoing study. Data were collected between July 15 and October 9, 2020. All materials were written in the Spanish language. After completing the survey, participants watched an informational video and received a list of relevant local resources and a code to redeem a $50 online gift card. The institutional review board of the Medical College of Wisconsin approved the study procedures and materials. Because names were not recorded during participant screening or survey data collection, the study was granted a waiver of written informed consent by the institutional review board. Eligible participants received a study informational letter via email or text message. Study staff reviewed the letter with participants and elicited their verbal informed consent.

The aims of the parent study were to examine the association of actual and perceived immigration laws and concerns with Latinx immigrants’ willingness to use services for HIV testing and for 2 factors associated with HIV infection: substance use disorders and intimate partner violence. Legal scans, expert interviews, and focus groups comprising diverse Latinx immigrants informed the construction of a de novo scale assessing immigration concerns that were associated with these study outcomes. The survey included measures assessing participants’ willingness to use services for HIV testing, substance use disorders, and intimate partner violence and, when appropriate, their actual use of these services. Additional measures relevant to study outcomes (eg, awareness of services and perceived stigma) were also included. The parent study was conducted in 4 US metropolitan areas with diverse immigration environments: Chicago, Illinois; Los Angeles, California; Phoenix, Arizona; and Raleigh-Durham, North Carolina. The data presented in this article were collected from participants in Chicago, Los Angeles, and Phoenix.

The design of the parent study, which comprises a cross-sectional survey of a nonprobability sample, was chosen to assess actual and, importantly, perceived immigration-associated risk among immigrant participants. All participants opted into study participation by contacting study staff. Considerable effort was dedicated to protecting participants’ anonymity. Names were not recorded during participant screening and survey data collection. Links to the self-administered survey were sent to eligible participants via text or email. Participants were informed that they could create a temporary email account for this purpose. To receive their online gift card, participants provided study staff with a randomly generated code displayed on the final screen of their survey. Mobile phone numbers and email and IP addresses were deleted after participants received their gift card code.

### Sample

Participants were adult, Spanish-speaking, noncitizen Latinx immigrants (with either documented or undocumented immigration status) who had lived in the US for at least 6 months (to ensure they had the opportunity to become familiar with US immigration laws and policies) and were currently living in Chicago, Los Angeles, or Phoenix. Additional eligibility criteria, including HIV-negative or unknown status and being sexually active, were used to examine parent study outcomes. A total of 379 individuals were sent survey links. Of those, 336 individuals (88.7% participation rate, as calculated using American Association of Public Opinion Research (AAPOR) guidelines for a nonrandom online access panel^7^) returned completed surveys, and 43 individuals did not. An additional 213 individuals contacted us but were deemed ineligible after screening.

### Participant Recruitment

Participants were recruited through social media, word of mouth, and community-based organizations. Study recruitment flyers were distributed widely in places visited by Latinx immigrants during the COVID-19 pandemic (eg, local ethnic markets, places where day laborers congregate, and school lunch pick-up sites) as well as through Facebook postings and email messages to organizations serving Latinx immigrants. Recruitment flyers invited adult Latinx immigrants to call a phone number to receive screening for eligibility to participate in a confidential survey study of immigrant health. Recruitment materials were provided in the Spanish language.

### Measures

Sociodemographic variables included age, national origin, language preference, health insurance status, and immigration documentation status (permanent resident, visa holder, immigrant with a work permit, undocumented immigrant, or other). Participant gender was self-identified as female, male, transgender, or other. Participant ethnicity was self-identified with a single item: “Are you Hispanic or Latino?” Response options were dichotomous, with 1 indicating yes and 0 indicating no. Participants were asked to indicate the total number of years and months they had lived in the US.

The 25 COVID-19 survey items elicited participants’ agreement or disagreement with statements on topics ranging from immigrants’ eligibility for COVID-19–related testing and treatment services (eg, “Hospital emergency departments are the only places immigrants who don’t have insurance can go to receive testing or medical care for COVID-19”) to public charge rules pertaining to immigrants’ financial independence and use of government-funded services (eg, “An immigrant who has used low-cost public medical services for COVID-19 may not be allowed to renew or advance their immigration status based on financial need”). Only 1 item (“All immigrants can receive publicly funded medical care for COVID-19 regardless of their immigration status”) was unequivocally true. Participants indicated their responses using a 4-point Likert-type scale, with 1 indicating strongly disagree and 4 indicating strongly agree. The survey items and response frequencies are presented in Table 1.

**Table 1.** Agreement Response Frequencies for Selected COVID-19 Survey Items Pertaining to Immigration Law

Item	Participants with agreement responses, No. (%) (N = 336)
Immigrants’ COVID-19 test results are reported to immigration authorities	66 (19.6)
It is better for immigrants to avoid being tested for COVID-19 because their medical records are available to immigration authorities	54 (16.1)
Doctors send a report to immigration authorities whenever an immigrant seeks medical care for COVID-19	58 (17.3)
Doctors must inform immigration officials if they believe that an immigrant has an infectious condition like COVID-19	46 (13.7)
Undocumented immigrants might be identified by immigration officials if they seek medical care for COVID-19	55 (16.4)
If an immigrant needs to be tested for COVID-19, it is best not to provide personal information because it will draw attention to his or her immigration status	69 (20.5)
Using publicly funded medical care for COVID-19 can make immigration authorities notice one’s immigration status	92 (27.4)
To avoid government attention, it is better for immigrants not to be tested or treated for COVID-19	52 (15.5)
Immigrants’ immigration prospects can be hurt if they get COVID-19 because immigration authorities will think that they didn’t follow self-quarantine rules	82 (24.4)
Being diagnosed with COVID-19 will make an immigrant less attractive as a US resident to immigration officials	77 (22.9)
Immigrants who become sick with COVID-19 will hurt their opportunities to adjust their status because immigration authorities may consider them undesirable citizens	69 (20.5)
Immigration officials may deny the applications of immigrants who have had COVID-19 because they think that people who have had COVID-19 were reckless and put others at risk	66 (19.6)
If an immigrant uses public medical services because they think they may have COVID-19, the government may make them pay back the cost of their treatment	135 (40.2)
Immigrants who are permanent residents (have green cards) should not apply for unemployment if they lost their employment due to COVID-19 because they will be a burden to the government	93 (27.7)
Immigrants who hope to regularize their immigration status should not go to publicly funded clinics for COVID-19 testing or treatment because immigration officials will think they cannot financially support themselves	106 (31.5)
An immigrant who has used low-cost public medical services for COVID-19 may not be allowed to renew or advance their immigration status based on financial need	96 (28.6)
Immigrants must have proof that they are legal residents to be eligible for low-cost or free medical treatment for COVID-19	80 (23.8)
Most medical providers and clinics require patients to present a valid state ID to receive services for COVID-19; if you don’t have a valid ID, they will not treat you	117 (34.8)
Hospital emergency departments are the only places immigrants who don’t have insurance can go to receive testing or medical care for COVID-19	89 (26.5)
All immigrants can receive publicly funded medical care for COVID-19 regardless of their immigration status^1^	223 (66.4)
Immigration authorities will use any excuse to deny an immigration petition of someone who has had COVID-19	137 (40.8)
Whether a record of having COVID-19 will be a problem when you submit an immigration petition or renew your residency depends on the immigration worker who reviews it	108 (32.1)
Immigration officials blame immigrants for COVID-19 and will look for excuses to deny their immigration applications	121 (36.0)
In the current political climate, it is wise for immigrants to avoid seeking medical care for COVID-19 because they may be deported	76 (22.6)
Immigration authorities can do whatever they want to immigrant communities now that there is COVID-19	78 (23.2)

Two items adapted from the Hispanic Stress Inventory^8^ assessed participants’ deportation experiences: “Have you ever been deported?” and “Has a family member or close friend ever been deported?” Willingness to identify a potentially exposed, undocumented immigrant during public health contact tracing was assessed with 2 items: “If you had COVID-19 and public health officials asked you for the names of people you had been around that might have been exposed, would you give them the name of someone you live with who was undocumented?” and “If you had COVID-19 and public health officials asked you for the names of people you had been around that might have been exposed, would you give them the name of someone you work closely with who was undocumented?” Responses to these items were dichotomous, with 1 indicating yes and 0 indicating no. The COVID-19–related items were developed relatively early in the pandemic (April-May 2020). Participants were not asked about their use of COVID-19–related testing and treatment services or whether they or anyone close to them had been diagnosed with COVID-19.

### Statistical Analysis

Deportation experience was dichotomized, with 1 indicating ever having been deported and/or ever having had a family member or close friend who was deported and 0 indicating never having been deported and never having had a family member or close friend who was deported. Responses regarding immigration status were aggregated into 1 (having any documented immigration status) and 0 (not having any documented immigration status). Likert-type responses were dichotomized, with 1 indicating agree and 0 indicating disagree. Time lived in the US was calculated in years and dichotomized into 1 (lived in the US for ≤5 years) and 0 (lived in the US for >5 years). All data analyses were performed using IBM SPSS Statistics, version 27 (IBM Corp).^9^

Descriptive statistics, including means and frequencies, were computed. Independent sample *t* tests (unpaired and 2-sided) were conducted to compare COVID-19 immigration survey items with participant gender, health insurance status, immigration status, deportation experiences, and years lived in the US.

Because willingness to identify a potentially exposed, undocumented immigrant during public health contact tracing was likely to be associated with perceived immigration risk, the contact tracing items were selected for further analysis. The items were aggregated, with 1 indicating willingness to identify an undocumented household member and/or coworker and 0 indicating unwillingness to identify an undocumented household member and/or coworker. Kuder-Richardson 20 correlations and 95% CIs were computed between the aggregated contact tracing variable and variables capturing perceived immigration risk (ie, immigration documentation status, deportation experiences, and years lived in the US). The significance threshold was set at *P* < .05.

Missing data were imputed using the multiple imputation module of IBM SPSS Statistics, version 27. Inspection of missing data patterns indicated a range of missing data from 0.3% to 22.0%. Diagnostic plots were constructed and examined. Comparisons of missing data patterns by demographic characteristics did not reveal significant associations. A total of 40 imputed data sets were computed in 840 iterations. The estimates from the imputed data sets were combined for the analyses.^10^

## Results

Among 336 adult Latinx immigrants, the mean (SD) age was 39.7 (8.9) years. Most participants (210 individuals [62.5%]) identified as female; 1 participant (0.3%) identified as transgender, and 1 participant (0.3%) identified as other. Most participants were born in Mexico (291 individuals [86.6%]) and preferred to speak Spanish all or most of the time (265 individuals [78.9%]). All participants self-identified as Hispanic or Latino. Eighty participants (23.8%) had health insurance. Only 22 participants (6.6%) had been deported, but more than one-third (128 participants [38.1%]) reported that a family member or close friend had been deported. Thirty-three participants (9.8%) had lived in the US for 5 years or less. Nearly two-thirds of participants (216 individuals [64.3%]) reported that their immigration status was undocumented. Additional sociodemographic characteristics are presented in Table 2.

**Table 2.** Participant Characteristics

Characteristic	Participants, No. (%) (N = 336)
Gender	
Male	124 (36.9)
Female	210 (62.5)
Transgender	1 (0.3)
Other	1 (0.3)
Age range, y	
18-24	18 (5.4)
25-44	221 (65.8)
45-54	84 (25.0)
55-64	9 (2,7)
≥65	4 (1.2)
Marital status	
Married	184 (54.8)
Long-term relationship but not married	106 (31.5)
Single	46 (13.7)
Educational level	
≤High school	248 (73.8)
Technical degree	29 (8.6)
Some college or college graduate	58 (17.3)
Monthly income, $	
0-999	164 (48.8)
1000-1999	118 (35.1)
2000-4999	52 (15.5)
≥5000	2 (0.6)
Health insurance status	
No	256 (76.2)
Yes	80 (23.8)
Immigration status	
Undocumented	216 (64.3)
Documented	120 (35.7)
Country of birth	
Cuba	2 (0.6)
El Salvador	8 (2.4)
Ecuador	3 (0.9)
Guatemala	14 (4.2)
Honduras	4 (1.2)
Mexico	291 (86.6)
Nicaragua	1 (0.3)
Other	13 (3.9)
Preferred language	
English always or most of the time	9 (2.7)
Both English and Spanish equally	60 (17.9)
Spanish always or most of the time	265 (78.9)
Another language	2 (0.6)

A substantial proportion of participants endorsed 1 or more statements about restrictions on immigrants’ access to COVID-19–related testing and treatment. A total of 80 participants (23.8%) agreed that immigrants must have proof of legal residency to be eligible for low-cost or free treatment for COVID-19, and 89 participants (26.5%) agreed that hospital emergency departments were the only source for COVID-19–related testing and treatment for uninsured immigrants. More than one-third of participants (117 individuals [34.8%]) believed that most medical providers would deny COVID-19 care if an immigrant did not have valid state identification.

Of particular concern was the number of participants who believed that using COVID-19 health care services could result in serious immigration consequences. In total, 92 participants (27.4%) agreed that using public services for COVID-19–related testing and treatment could jeopardize an individual’s immigration prospects by drawing attention to immigration status, and 106 participants (31.6%) agreed that using public COVID-19–related testing and treatment services could raise questions about an immigrant’s financial standing. A substantial proportion of participants believed that simply receiving a diagnosis of COVID-19 could have negative immigration consequences. A total of 69 participants (20.5%) agreed that immigration authorities may consider immigrants who have been diagnosed with COVID-19 to be undesirable citizens, and 121 participants (36.0%) agreed that immigration officials blame immigrants for COVID-19 and will look for excuses to deny their immigration applications. A total of 52 participants (15.5%) agreed that, to avoid government attention, it is better for immigrants not to be tested or treated for COVID-19. Additional COVID-19 survey items and response frequencies are presented in Table 2.

Independent sample *t* tests examining mean participant agreement with COVID-19 survey items yielded no statistically significant differences based on participant gender or health insurance status. However, significant differences were observed between mean responses to several items by immigration documentation status, time lived in the US, and deportation experiences. The mean agreement responses of documented and undocumented immigrants varied significantly on only 2 items, and *t* tests comparing item responses revealed that documented immigrants had higher agreement with the following 2 items: “Hospital emergency departments are the only places immigrants who don’t have insurance can go to receive testing or medical care for COVID-19” (mean [SD] agreement, 1.61 [1.02] among undocumented immigrants vs 2.04 [1.15] among documented immigrants; difference in mean agreement, −0.42 [95% CI, −0.67 to −0.17]; *t* = −3.34; *P* = .001) and “Immigration authorities can do whatever they want to immigrant communities now that there is COVID-19” (mean [SD] agreement, 1.52 [0.93] among undocumented immigrants vs 1.88 [1.14] among documented immigrants; difference in mean agreement, −0.36 [95% CI, −0.59 to −0.12]; *t* = −3.02; *P* = .003).

Comparisons of mean agreement responses as a function of years lived in the US (≤5 years vs >5 years) indicated statistically significant differences in mean agreement with 18 of the 25 items. Agreement among recent immigrants was higher for almost all of these items. Notably, the mean agreement of item responses associated with the routine release of medical information to immigration authorities was consistently higher among recent immigrants. For example, regarding the item, “Immigrants’ COVID-19 test results are reported to immigration authorities,” the mean (SD) agreement among immigrants living in the US for 5 years or less was 2.10 (1.19) vs 1.47 (0.95) among immigrants living in the US for more than 5 years (difference in mean agreement, 0.63 [95% CI, 0.28-0.96]; *t* = 3.62; *P* = .001).

Comparisons of mean agreement responses by participant deportation experiences revealed significant differences in 10 items. In every instance, the mean item agreement of those with deportation experiences was higher compared with those who did not have deportation experiences. Participants with deportation experiences had higher endorsement of 1 survey item reflecting the unpredictable nature of immigration processes: “Whether a record of having COVID-19 will be a problem when you submit an immigration petition or renew your residency depends on the immigration worker who reviews it.” The mean (SD) agreement for this item was 1.73 (1.01) among those without deportation experiences vs 2.13 (1.22) among those with deportation experiences (difference in mean agreement, −0.40 [95% CI, −0.66 to −0.13]; *t* = −2.98; *P* = .003). Additional comparisons of mean agreement responses are shown in Table 3, Table 4, and Table 5.

**Table 3.** Mean Agreement With COVID-19 Survey Items by Immigration Status

Item	Participant agreement, mean (SD)^a^		Difference in mean agreement (95% CI)	*t*
	Undocumented immigration status	Documented immigration status		
Immigrants’ COVID-19 test results are reported to immigration authorities	1.47 (0.96)	1.63 (1.05)	−0.16 (−0.37 to 0.04)	−1.54
It is better for immigrants to avoid being tested for COVID-19 because their medical records are available to immigration authorities	1.45 (0.96)	1.54 (1.02)	−0.09 (−0.32 to 0.13)	−0.83
Doctors send a report to immigration authorities whenever an immigrant seeks medical care for COVID-19	1.46 (0.88)	1.58 (1.02)	−0.11 (−0.34 to 0.10)	−1.02
Doctors must inform immigration officials if they believe that an immigrant has an infectious condition like COVID-19	1.38 (0.75)	1.54 (1.07)	−0.16 (−0.37 to 0.04)	−1.55
Undocumented immigrants might be identified by immigration officials if they seek medical care for COVID-19	1.46 (0.83)	1.51 (0.83)	−0.05 (−0.24 to 0.14)	−0.52
If an immigrant needs to be tested for COVID-19, it is best not to provide personal information because it will draw attention to his or her immigration status	1.57 (1.11)	1.71 (1.27)	−0.14 (−0.40 to 0.11)	−1.10
Using publicly funded medical care for COVID-19 can make immigration authorities notice one’s immigration status	1.78 (1.60)	1.77 (1.52)	0 (−0.30 to 0.32)	0.04
To avoid government attention, it is better for immigrants to not be tested or treated for COVID-19	1.40 (0.91)	1.49 (0.87)	−0.08 (−0.30 to 0.12)	−0.81
Immigrants’ immigration prospects can be hurt if they get COVID-19 because immigration authorities will think that they didn’t follow self-quarantine rules	1.78 (1.60)	1.66 (1.52)	0.12 (−0.22 to 0.46)	0.74
Being diagnosed with COVID-19 will make an immigrant less attractive as a US resident to immigration officials	1.55 (1.29)	1.70 (2.07)	−0.14 (−0.42 to 0.13)	−1.04
Immigrants who become sick with COVID-19 will hurt their opportunities to adjust their status because immigration authorities may consider them undesirable citizens	1.57 (1.94)	1.59 (0.91)	−0.02 (−0.30 to 0.25)	−0.17
Immigration officials may deny the applications of immigrants who have had COVID-19 because they think that people who have had COVID-19 were reckless and put others at risk	1.53 (0.91)	1.63 (0.91)	−0.09 (−0.30 to 0.11)	−0.90
If an immigrant uses public medical services because they think they may have COVID-19, the government may make them pay back the cost of their treatment	2.04 (1.68)	2.07 (2.42)	−0.03 (−0.41 to 0.34)	−0.18
Immigrants who are permanent residents (have green cards) should not apply for unemployment if they lost their employment due to COVID-19 because they will be a burden to the government	1.73 (1.50)	1.83 (1.43)	−0.09 (−0.38 to 0.20)	−0.64
Immigrants who hope to regularize their immigration status should not go to publicly funded clinics for COVID-19 testing or treatment because immigration officials will think they cannot financially support themselves	1.88 (1.83)	1.97 (1.82)	−0.08 (−0.44 to 0.27)	−0.48
An immigrant who has used low-cost public medical services for COVID-19 may not be allowed to renew or advance their immigration status based on financial need	1.84 (1.60)	1.87 (1.55)	−0.03 (−0.43 to 0.28)	−0.21
Immigrants must have proof that they are legal residents to be eligible for low-cost or free medical treatment for COVID-19	1.64 (1.08)	1.87 (1.55)	−0.23 (−0.50 to 0.02)	−1.78
Most medical providers and clinics require patients to present a valid state ID to receive services for COVID-19; if you don’t have a valid ID, they will not treat you	1.95 (2.07)	2.20 (1.85)	−0.25 (−0.61 to 0.09)	−1.49
Hospital emergency departments are the only places immigrants who don’t have insurance can go to receive testing or medical care for COVID-19^b^	1.61 (1.02)	2.04 (1.15)	−0.42 (−0.67 to −0.17)	−3.34
All immigrants can receive publicly funded medical care for COVID-19 regardless of their immigration status^1^	3.08 (1.46)	2.81 (1.58)	0.27 (0 to 0.55)	−1.54
Immigration authorities will use any excuse to deny an immigration petition of someone who has had COVID-19^c^	2.06 (2.03)	2.13 (2.17)	−0.06 (−0.45 to 0.31)	−0.83
Whether a record of having COVID-19 will be a problem when you submit an immigration petition or renew your residency depends on the immigration worker who reviews it	1.84 (1.71)	1.97 (1.55)	−0.13 (−0.43 to 0.17)	−0.85
Immigration officials blame immigrants for COVID-19 and will look for excuses to deny their immigration applications	1.92 (1.68)	2.04 (1.72)	−.012 (−.043 to 0.19)	−1.55
In the current political climate, it is wise for immigrants to avoid seeking medical care for COVID-19 because they may be deported	1.62 (1.05)	1.75 (1.05)	−0.13 (−0.36 to 0.09)	−0.52
Immigration authorities can do whatever they want to immigrant communities now that there is COVID-19^c^	1.52 (0.93)	1.88 (1.14)	−0.36 (−0.59 to −0.12)	−3.02

^a^ Responses were based on a 4-point Likert-type scale, with 1 indicating strongly disagree and 4 indicating strongly agree.

^b^ *P* = .001.

^c^ *P* = .003.

**Table 4.** Mean Agreement With COVID-19 Survey Items by Years Lived in the US

Item	Participant agreement, mean (SD)^a^		Difference in mean agreement (95% CI)	*t*
	≤5 y in US	>5 y in US		
Immigrants’ COVID-19 test results are reported to immigration authorities^b^	2.10 (1.19)	1.47 (0.95)	0.63 (0.28 to 0.96)	3.62
It is better for immigrants to avoid being tested for COVID-19 because their medical records are available to immigration authorities^c^	1.82 (1.20)	1.45 (0.88)	0.37 (0.03 to 0.71)	2.17
Doctors send a report to immigration authorities whenever an immigrant seeks medical care for COVID-19^d^	1.93 (1.06)	1.46 (0.85)	0.47 (0.15 to 0.79)	2.87
Doctors must inform immigration officials if they believe that an immigrant has an infectious condition like COVID-19^e^	1.89 (1.12)	1.39 (0.88)	0.49 (0.17 to 0.81)	3.06
Undocumented immigrants might be identified by immigration officials if they seek medical care for COVID-19^f^	1.88 (1.17)	1.44 (0.81)	0.44 (0.11 to 0.76)	2.68
If an immigrant needs to be tested for COVID-19, it is best not to provide personal information because it will draw attention to his or her immigration status	1.91 (1.21)	1.59 (1.05)	0.31 (−0.04 to 0.67)	1.72
Using publicly funded medical care for COVID-19 can make immigration authorities notice one’s immigration status^g^	2.27 (1.37)	1.72 (1.20)	0.55 (0.16 to 0.94)	2.77
To avoid government attention, it is better for immigrants to not be tested or treated for COVID-19^b^	1.96 (1.33)	1.38 (0.78)	0.58 (0.25 to 0.90)	3.52
Immigrants’ immigration prospects can be hurt if they get COVID-19 because immigration authorities will think that they didn’t follow self-quarantine rules^h^	2.18 (1.48)	1.68 (1.09)	0.50 (0.08 to 0.91)	2.39
Being diagnosed with COVID-19 will make an immigrant less attractive as a US resident to immigration officials^c^	1.99 (1.49)	1.57 (1.09)	0.42 (0.02 to 0.81)	2.11
Immigrants who become sick with COVID-19 will hurt their opportunities to adjust their status because immigration authorities may consider them undesirable citizens^g^	2.04 (1.40)	1.53 (0.88)	0.51 (0.15 to 0.87)	2.78
Immigration officials may deny the applications of immigrants who have had COVID-19 because they think that people who have had COVID-19 were reckless and put others at risk^h^	1.95 (1.21)	1.53 (0.85)	0.42 (0.09 to 0.76)	2.50
If an immigrant uses public medical services because they think they may have COVID-19, the government may make them pay back the cost of their treatment	2.26 (1.71)	2.02 (1.44)	0.23 (−0.24 to 0.71)	0.98
Immigrants who are permanent residents (have green cards) should not apply for unemployment if they lost their employment due to COVID-19 because they will be a burden to the government^e^	2.36 (1.58)	1.71 (1.16)	0.65 (0.24 to 1.05)	3.14
Immigrants who hope to regularize their immigration status should not go to publicly funded clinics for COVID-19 testing or treatment because immigration officials will think they cannot financially support themselves^c^	2.31 (1.29)	1.87 (1.31)	0.43 (0.02 to 0.85)	2.07
An immigrant who has used low-cost public medical services for COVID-19 may not be allowed to renew or advance their immigration status based on financial need^c^	2.23 (1.21)	1.81 (1.31)	0.42 (0.02 to 0.85)	2.08
Immigrants must have proof that they are legal residents to be eligible for low-cost or free medical treatment for COVID-19^i^	2.26 (1.33)	1.67 (1.12)	0.58 (0.20 to 0.96)	3.02
Most medical providers and clinics require patients to present a valid state ID to receive services for COVID-19; if you don’t have a valid ID, they will not treat you	2.27 (1.40)	2.02 (1.61)	0.25 (−0.20 to 0.70)	1.08
Hospital emergency departments are the only places immigrants who don’t have insurance can go to receive testing or medical care for COVID-19^d^	2.33 (1.33)	1.72 (1.20)	0.60 (0.19 to 1.00)	2.91
All immigrants can receive publicly funded medical care for COVID-19 regardless of their immigration status^1^	2.65 (1.32)	3.01 (1.48)	−0.36 (−0.80 to 0.08)	−1.60
Immigration authorities will use any excuse to deny an immigration petition of someone who has had COVID-19	2.40 (1.59)	2.06 (1.48)	0.33 (−0.08 to 0.76)	1.55
Whether a record of having COVID-19 will be a problem when you submit an immigration petition or renew your residency depends on the immigration worker who reviews it	2.21 (1.40)	1.85 (1.52)	0.35 (−0.06 to 0.78)	1.64
Immigration officials blame immigrants for COVID-19 and will look for excuses to deny their immigration applications	2.27 (1.48)	1.93 (1.44)	0.33 (−0.08 to 0.76)	1.55
In the current political climate, it is wise for immigrants to avoid seeking medical care for COVID-19 because they may be deported	2.06 (1.33)	1.93 (0.98)	0.42 (0.06 to 0.79))	2.29
Immigration authorities can do whatever they want to immigrant communities now that there is COVID-19^i^	2.18 (1.38)	1.61 (1.01)	0.57 (0.20 to 0.94)	−3.02

^a^ Responses were based on a 4-point Likert-type scale, with 1 indicating strongly disagree and 4 indicating strongly agree.

^b^ *P* = .001.

^c^ *P* = .03.

^d^ *P* = .004.

^e^ *P* = .002.

^f^ *P* = .007.

^g^ *P* = .006.

^h^ *P* = .01.

^i^ *P* = .003.

**Table 5.** Mean Agreement With COVID-19 Survey Items by Deportation Experience

Item	Participant agreement, mean (SD)^a^		Difference in mean agreement (95% CI)	*t*
	No deportation experience	Deportation experience		
Immigrants’ COVID-19 test results are reported to immigration authorities^b^	1.44 (1.15)	1.68 (0.93)	−0.24 (−0.46 to 0.007)	−2.03
It is better for immigrants to avoid being tested for COVID-19 because their medical records are available to immigration authorities	1.42 (1.05)	1.58 (0.80)	−0.16 (−0.36 to 0.05)	−1.47
Doctors send a report to immigration authorities whenever an immigrant seeks medical care for COVID-19	1.43 (1.80)	1.62 (0.75)	−0.19 (−0.40 to 0.01)	−1.83
Doctors must inform immigration officials if they believe that an immigrant has an infectious condition like COVID-19	1.40 (0.87)	1.50 (1.02)	−0.10 (−0.32 to 0.11)	−0.93
Undocumented immigrants might be identified by immigration officials if they seek medical care for COVID-19^c^	1.35 (1.15)	1.68 (0.56)	−0.33 (−0.52 to −0.12)	−3.24
If an immigrant needs to be tested for COVID-19, it is best not to provide personal information because it will draw attention to his or her immigration status	1.59 (1.17)	1.67 (1.02)	−0.08 (−0.31 to 0.15)	−0.68
Using publicly funded medical care for COVID-19 can make immigration authorities notice one’s immigration status^d^	1.64 (1.36)	1.99 (1.13)	−0.35 (−0.59 to −0.10)	−2.79
To avoid government attention, it is better for immigrants to not be tested or treated for COVID-19	1.39 (0.94)	1.50 (0.80)	−0.11 (−0.31 to 0.09)	−1.07
Immigrants’ immigration prospects can be hurt if they get COVID-19 because immigration authorities will think that they didn’t follow self-quarantine rules	1.70 (1.22)	1.78 (1.20)	−0.08 (−0.32 to 0.16)	−0.63
Being diagnosed with COVID-19 will make an immigrant less attractive as a US resident to immigration officials^e^	1.50 (1.25)	1.77 (1.07)	−0.27 (−0.50 to −0.02)	−2.16
Immigrants who become sick with COVID-19 will hurt their opportunities to adjust their status because immigration authorities may consider them undesirable citizens	1.53 (1.03)	1.66 (0.96)	−0.13 (−0.36 to 0.09)	−1.19
Immigration officials may deny the applications of immigrants who have had COVID-19 because they think that people who have had COVID-19 were reckless and put others at risk^f^	1.47 (1.10)	1.72 (0.80)	−0.25 (−0.46 to −0.02)	−2.22
If an immigrant uses public medical services because they think they may have COVID-19, the government may make them pay back the cost of their treatment^e^	1.91 (1.82)	2.25 (1.53)	−0.34 (−0.64 to −0.03)	−2.21
Immigrants who are permanent residents (have green cards) should not apply for unemployment if they lost their employment due to COVID-19 because they will be a burden to the government	1.72 (1.17)	1.84 (1.32)	−0.12 (−0.38 to 0.14)	−0.89
Immigrants who hope to regularize their immigration status should not go to publicly funded clinics for COVID-19 testing or treatment because immigration officials will think they cannot financially support themselves	1.85 (1.49)	2.01 (1.39)	−0.16 (−0.43 to 0.11)	−1.16
An immigrant who has used low-cost public medical services for COVID-19 may not be allowed to renew or advance their immigration status based on financial need	1.75 (1.47)	2.01 (1.36)	−0.26 (−0.52 to 0.004)	−1.96
Immigrants must have proof that they are legal residents to be eligible for low-cost or free medical treatment for COVID-19	1.63 (1.28)	1.88 (1.32)	−0.25 (−0.50 to 0.01)	−1.91
Most medical providers and clinics require patients to present a valid state ID to receive services for COVID-19; if you don’t have a valid ID, they will not treat you	1.79 (1.67)	2.22 (1.65)	−0.43 (−0.59 to 0.01)	−1.86
Hospital emergency departments are the only places immigrants who don’t have insurance can go to receive testing or medical care for COVID-19	1.79 (1.41)	1.76 (1.42)	0.03 (−0.24 to 0.31)	0.23
All immigrants can receive publicly funded medical care for COVID-19 regardless of their immigration status^1^	3.05 (1.70)	2.86 (1.67)	0.19 (−0.11 to 0.48)	1.21
Immigration authorities will use any excuse to deny an immigration petition of someone who has had COVID-19	1.58 (1.82)	1.80 (1.60)	−0.22 (−0.46 to 0.01)	−1.88
Whether a record of having COVID-19 will be a problem when you submit an immigration petition or renew your residency depends on the immigration worker who reviews it^g^	1.73 (1.01)	2.13 (1.22)	−0.40 (−0.66 to −0.13)	−2.98
Immigration officials blame immigrants for COVID-19 and will look for excuses to deny their immigration applications^d^	1.81 (1.73)	2.20 (1.29)	−0.39 (−0.66 to −0.12)	−2.85
In the current political climate, it is wise for immigrants to avoid seeking medical care for COVID-19 because they may be deported^f^	1.57 (1.22)	1.83 (0.99)	−0.26 (−0.49 to −0.02)	−2.21
Immigration authorities can do whatever they want to immigrant communities now that there is COVID-19	1.58 (1.28)	1.80 (0.99)	−0.22 (−0.46 to 0.01)	−1.88

^a^ Responses were based on a 4-point Likert-type scale, with 1 indicating strongly disagree and 4 indicating strongly agree.

^b^ *P* = .04.

^c^ *P* = .001.

^d^ *P* = .005.

^e^ *P* = .03.

^f^ *P* = .02.

^g^ *P* = .003.

With regard to contact tracing, 96 participants (28.6%) and 114 participants (33.9%), respectively, reported that they would be unwilling to provide public health representatives with the name of a potentially exposed, undocumented household member or coworker. Willingness to provide names for contact tracing was inversely associated with having deportation experiences (*r* = −0.17; 95% CI, −0.06 to 0.27; *P* = .003). Participants with deportation experiences more frequently reported that they would be unwilling to identify an undocumented contact compared with participants without deportation experiences. Reluctance to identify an undocumented contact was not significantly associated with years lived in the US (*r* = 0.07; 95% CI, −0.16 to 0.17; *P* = .15) or immigration status (*r* = 0.03; 95% CI, −0.07 to 0.13; *P* = .56).

## Discussion

In this survey study, a substantial number of immigrants endorsed statements (which were often erroneous) about restrictions on immigrants’ access to COVID-19–related testing and treatment services and the potential negative immigration ramifications of using these services. Most participants had no health insurance, and many believed that if they used publicly funded health care services, they would draw attention to their immigration status or jeopardize their immigration prospects. Assumptions about ineligibility for COVID-19–related testing or treatment and/or fears about the immigration ramifications of using these services may lead immigrants to avoid or delay critical care.

Comparisons of mean responses on the COVID-19–related items identified particularly vulnerable immigrant groups. Living in the US for 5 years or less was associated with greater agreement on most of the COVID-19 items compared with living in the US for more than 5 years. Notably, recent immigrants had higher endorsement of statements associated with the confidentiality of their medical information. This finding may be attributable to recent immigrants’ not having had the opportunity to learn about their rights in the US. Language barriers may make it even more difficult for them to become familiar with US laws.

Having deportation experiences was associated with greater agreement with several COVID-19 items compared with not having deportation experiences. Notably, most participants with deportation experiences had not been deported themselves but instead reported that a family member or close friend had been deported. The impact of the deportation experience appeared to extend to those left behind. Furthermore, if individuals perceive that immigration laws are applied capriciously and are associated with poor outcomes, hesitancy to engage with public health professionals for COVID-19–related testing or to seek health care may increase.

Analyses of responses to COVID-19 items by documentation status revealed an unexpected pattern. Although much of the literature on immigrant health has focused on undocumented immigrants,^11,12,13^ item responses did not differ significantly between documented and undocumented immigrants. In addition, in 2 instances in which item responses did differ significantly by documentation status, endorsement of the items among documented immigrants was greater than that of undocumented immigrants. Given that the immigration cases of documented immigrants undergo formal review, these individuals may have been particularly concerned about being in conflict with immigration laws or authorities.

A substantial proportion of participants reported that they would not identify a potentially exposed, undocumented household member or coworker during contact tracing. Those with deportation experiences more frequently reported that they would not identify an undocumented contact compared with those without deportation experiences. These findings are consistent with the greater endorsement of many individual COVID-19 items among those with deportation experiences relative to those without deportation experiences. However, willingness to identify an undocumented contact did not differ significantly by participants’ documentation status. In this instance, the documentation status of the potentially exposed contact, rather than the status of the participant, may be most salient.

### Limitations

This study has limitations. Although data were collected across 3 US cities, surveys were completed by a nonrandom sample of Latinx immigrants, thereby limiting the generalizability of findings. The survey sample included more individuals who identified as female vs male, and only 2 participants identified as neither (1 transgender and 1 other). However, survey responses did not vary substantially by gender. The sample also included relatively few recent immigrants. Given recent immigrants’ higher mean agreement with many of the COVID-19 survey items compared with longer-term residents, additional attention to this subgroup is warranted. The COVID-19 survey items used in this study were exploratory. Additional research is needed to examine associations between item responses and COVID-19–related behaviors.

## Conclusions

The success of public health efforts to reduce SARS-CoV-2 infection rates and reduce COVID-19 morbidity and mortality depends on the cooperation of all community members. Optimizing the health of immigrants advances the health of the entire community. To the extent that immigration concerns deter immigrants from using services for COVID-19–related testing and treatment or from engaging in contact tracing, the success of efforts to manage the pandemic may be jeopardized. Results of this preliminary study highlight the need for collaborations among medical practitioners, legal professionals, and Latinx immigrants to address concerns. It is therefore important to encourage and expand recent inquiries into medical-legal partnerships.^11,12,13^

In addition, public health leaders may want to consider designing programs for COVID-19–related testing, contact tracing, and vaccine administration to allay immigration concerns. Messages about immigrants’ eligibility for services regardless of documentation status could be disseminated widely in Spanish, English, and other locally relevant languages. Alternatives to official state identification, for which undocumented immigrants may be ineligible, can be accepted at COVID-19–related testing, health care, and vaccination sites. Trusted information sources may be used to inform immigrants about their rights in the US, particularly regarding health care, confidentiality, and immigration issues. Documented immigrants, recent immigrants, and those who have experienced the deportation of a family member or friend could be included in these efforts.

Further research is needed on immigrants’ immigration concerns about public health contact tracing. To our knowledge, a quantitative inquiry has not been conducted to examine Latinx immigrants’ specific immigration concerns about contact tracing. Understanding marginalized individuals’ concerns about contact tracing is a necessary precursor to successful infectious disease intervention efforts.
